# Vedolizumab Is Associated with Longer Drug Sustainability Compared to Infliximab in Moderate-to-Severe Ulcerative Colitis: Long-Term Real-World Cohort Data

**DOI:** 10.3390/jcm12134488

**Published:** 2023-07-04

**Authors:** Tom Konikoff, Henit Yanai, Dror Libchik, Irit Avni-Biron, Yifat Snir, Hagar Banai, Yelena Broytman, Iris Dotan, Jacob E. Ollech

**Affiliations:** 1 Division of Gastroenterology, Rabin Medical Center, Petah Tikva 4941492, Israel; tomko@clalit.org.il (T.K.); henitya@clalit.org.il (H.Y.); irisdo@clalit.org.il (I.D.); 2 Sackler Faculty of Medicine, Tel Aviv University, Tel Aviv 6423906, Israel; 3 Faculty of Agriculture, Food and Environment, Hebrew University of Jerusalem, Rehovot 7610001, Israel

**Keywords:** biologics in UC, drug efficacy in UC, drug survival in UC

## Abstract

Background and Aim: Drug sustainability (DS) is a surrogate marker for treatment efficacy. We aimed to compare the DS of two main biologics used to treat moderate-to-severe ulcerative colitis (UC), infliximab (IFX) and vedolizumab (VDZ), in a real-world setting. Methods: We conducted a retrospective cohort study at a tertiary medical center in Israel. We included patients treated between 1 December 2017 and 1 May 2021, who were followed for up to 300 weeks. DS was defined as corticosteroid-, surgical-, and hospitalization-free treatment. Results: 217 patients with UC were included. VDZ had a significantly longer median DS of 265.6 weeks compared to IFX’s 106.5 weeks (*p* = 0.001) in treatment-naïve patients, even when adjusting for disease severity (HR 0.55 95 CI 0.3–0.98, *p* = 0.042). In treatment-experienced patients, DS was comparable between IFX and VDZ (*p* = 0.593). Conclusions: VDZ showed significantly longer DS in treatment-naïve patients with UC compared to IFX, also when adjusted for disease severity. There was no difference in DS between VDZ and IFX in treatment-experienced patients and patients switching from one drug to another. VDZ may be a suitable first-line treatment for biologic-naïve patients with moderate-to-severe UC.

## 1. Introduction

Ulcerative colitis (UC) is rising worldwide, reaching a prevalence of over 5 per 1000 people and 2 per 1000 people in Europe and the US, respectively [1]. This rise, along with the concomitant increase in life expectancy, means more patients will experience disease burden for a longer time. Therefore, long-term therapy is needed with both effective and safe drugs. The introduction of biologics in the past two decades has had a profound effect on the treatment landscape of UC. Nevertheless, many patients do not respond to therapy induction and lose response over time [2,3]. Although switching to the next biologic drug is possible, loss-of-response rates are even higher for biologic-experienced patients [4]. This is largely believed to be due to the combination of immunogenicity of these drugs and the metabolic- and immune-related factors of the patient. Therapeutic goals have also undergone significant evolution as the therapeutic options have advanced. These endpoints are usually developed by expert consensuses and are based on outcomes of controlled studies. However, the real-world setting often differs greatly from the controlled setting and different patient-specific cases may not be represented in the latter. Drug sustainability (DS), sometimes referred to as “drug retention” or “drug persistence”, has been suggested as a reliable endpoint in the real-world setting [5]. Hence, it is important to evaluate DS, both as a clinical target and as a surrogate marker for drug efficacy, especially when choosing the first biologic. This is particularly important as head-to-head studies of biologic drugs in inflammatory bowel disease (IBD) are scarce. Unfortunately, data regarding DS are limited, often derived from clinical-phase drug studies that substantially differ from real-world clinical settings [6]. Therefore, we aimed to assess the DS of two biologic drugs used to treat UC: infliximab (IFX) and vedolizumab (VDZ).

## 2. Materials and Methods

This retrospective cohort study was conducted at the Rabin Medical Center, a large tertiary referral center in Israel.

### 2.1. Patient Selection and Follow-Up Period

We included patients 18 years old and above who received IFX or VDZ at the RMC infusion center between 1 December 2017 and 1 May 2021. Patients with acute severe colitis, Crohn’s disease, those with IBD-U, or those that underwent colectomy were excluded. Patients were identified using the combination of ICD-9 codes for UC diagnosis and the internal health maintenance operator (HMO) codes for IV treatment of the specific biologic drug. Patients were followed from the first documentation of therapy until the last follow-up date.

### 2.2. Outcomes

We assessed the DS of IFX and VDZ. DS was defined as long as corticosteroid-, surgical-, and hospitalization-free treatment was documented. We assessed DS in treatment-naïve and treatment-experienced patients, as well as in patients treated with one of the investigated drugs after failing the other.

### 2.3. Data Collection

An extensive medical chart review was performed. Patients’ data were collected from the first treatment documentation to the end of follow-up. Data collected included sex, age at diagnosis, age at treatment start, length of disease until treatment was started, disease extent (proctitis, left-sided or extensive), history of previous treatments with biologics, and comprehensive clinical laboratory data. We also documented if treatment was in combination with an immunomodulatory drug. We separately compared those previously naïve to biologic drugs and those who were experienced (second-line treatment) separately.

### 2.4. Statistical Analysis

We used the Fisher exact test for categorical variables and students’ T-test or Wilcoxon test for continuous variables when comparing the two groups and then constructed a Kaplan–Meier survival curve to depict comparative DS (depicted by length of drug treatment in weeks). We performed a univariate analysis for factors associated with treatment cessation (sustainability) to calculate the factor-specific hazard ratio (HR). We then adjusted for confounding factors (such as factors associated with disease severity, as described below in the Section 3) by performing a multivariate COX regression model and calculated the HR for DS. All results are reported at a 95% confidence limit. All statistical analysis was conducted using the SAS software version 9.4.

## 3. Results

### 3.1. Baseline Population Characteristics

**Figure 1:**
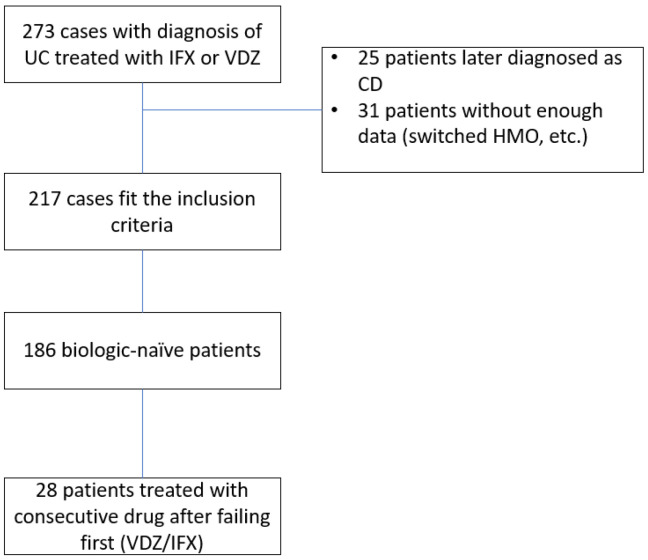
Study design. UC—Ulcerative colitis, CD—Crohn’s disease, HMO—health maintenance organization, IFX—infliximab, VDZ—vedolizumab.

A total of 273 patients were treated with IFX or VDZ at our center within the study time frame. Twenty-five patients were excluded due to a change in diagnosis, and 31 were excluded due to insufficient data (usually after switching HMO or treatment site or traveling abroad). The study design scheme is shown in Figure 1. A final 217 patients were included in the analysis, with a total follow-up period of 29,686 patient weeks. Of those, 186 were treatment-(biologic)-naïve, and of whom 28 were consequently treated with the second agent investigated after failing the first. Thus, 109 patients were treated with VDZ as the first drug, and 77 (41.3%) patients were treated with IFX as the first drug.

When comparing the two groups, more patients with proctitis were treated with VDZ (22% vs. 10.3%, *p* = 0.048). IFX was used more often with an immunomodulatory agent (14.2% vs. 2.7%, *p* = 0.004). There were also differences between the groups in baseline clinical features. Specifically, patients treated with IFX had lower baseline albumin (3.4 vs. 4.3, *p* = 0.001) and total protein (6.2 vs. 7.2, *p* ≤ 0.001) as well as a higher baseline calprotectin (4425 vs. 529, *p* = 0.001), baseline CRP (1.3 vs. 0.5, *p* = 0.002), and higher baseline neutrophil-to-lymphocyte ratio (4.2 vs. 2.7, *p* = 0.000). Patients treated with IFX also had lower baseline hemoglobin (median levels 11 vs. 13, *p* = 0.001) and baseline hematocrit levels (median 33.8 vs. 40.9, *p* = 0.001); the hemoglobin-to-hematocrit ratio was not different between the groups (*p* = 0.640). Baseline characteristics are shown in Table 1.

### 3.2. Drug Sustainability

**Figure 2:**
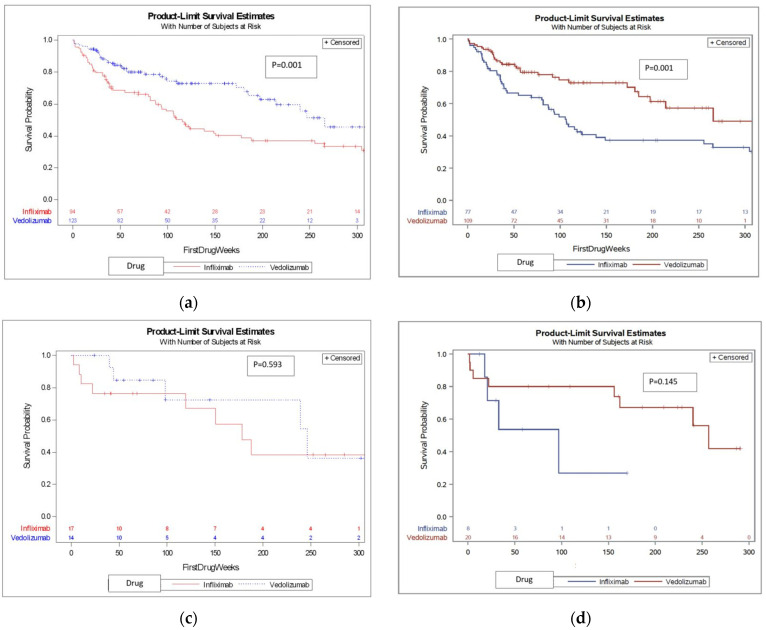
Survival curves of the DS of IFX and VDZ in different patient settings. (**a**) DS (treatment length, in weeks) of IFX and VDZ in the total cohort; (**b**) DS (treatment length, in weeks) of IFX and VDZ in treatment-naïve patient; (**c**) DS (treatment length, in weeks) of IFX and VDZ in treatment experienced patients; (**d**) Subsequent therapy DS (treatment length, in weeks) of IFX and VDZ.

We first compared treatment-naïve patients and found that VDZ was associated with a median survival time of 265.6 weeks (121.3–273.1), whereas IFX was associated with a significantly shorter median survival time of 106.5 (82.9–135) weeks (see KM survival curve shown in Figure 2, *p* = 0.001). We then compared treatment-experienced patients and found no difference in DS between IFX and VDZ (KM survival curve is shown in Figure 2c, *p* = 0.593). When combining the whole cohort (treatment naïve and experienced), VDZ was again associated with significantly longer drug survival (Figure 2, *p* = 0.001).

Due to differences in baseline parameters (described in Table 1 for treatment-naïve patients) between patients receiving IFX and VDZ, we further analyzed the cohort after adjusting for factors associated with disease severity, specifically for hemoglobin, hematocrit, neutrophil-to-lymphocyte ratio, albumin, CRP, and total protein, as well as other important treatment-related factors, such as age and combination therapy. This difference in DS between drugs (with vedolizumab having longer DS) was preserved after adjusting for these factors (HR 0.55 95 CI 0.3–0.98, *p* = 0.042). After adjustment, the type of drug (VDZ or IFX) was the only statistically significant factor associated with DS over time, as shown in Table 2.

### 3.3. Subsequent Treatment with a Second Drug

A total of 28 patients that failed either IFX (*n* = 8) or VDZ (*n* = 20) went on to be treated with the other drug (baseline characteristics of the two groups are found in Table S1 in the Supplementary Materials). When analyzing these patients, there was no significant difference in DS between IFX and VDZ (median 96.9 vs. 156.1, *p* = 0.145. KM survival curve shown in Figure 2d). See Table S2 (Supplementary Materials) for factors associated with DS in this subgroup.

## 4. Discussion

In this retrospective cohort study with detailed patient-level data, we aimed to assess the difference in DS between IFX and VDZ in the treatment of patients with UC.

We found that VDZ was associated with longer DS than IFX, even after correcting for multiple parameters associated with disease severity. In the absence of head-to-head comparisons, the retrospective evaluation of real-world data is an accepted approach for comparing drug efficacy. DS has been increasingly used as a surrogate for treatment efficacy when evaluating different therapeutic agents in a real-world setting [7,8,9]. It measures continuing medication prescription based on ongoing therapeutic efficacy. Importantly, it reflects real-world practice that allows for treatment optimization, including dose adjustments. The rate of failure and duration of DS for various drugs are different and provide important comparative efficacy data given the paucity of head-to-head trials of advanced therapies in UC [7,9]. DS data, in some respects, may be even more informative than head-to-head trials by more closely resembling real-world practice. Our study’s strengths included the large amount of patient-level data that we had for each patient, thus allowing for the assessment of baseline disease severity and the ability to control for these factors when comparing the two drugs. We also had a strict definition of DS, defined as long as patients were corticosteroid-, hospitalization-, and surgery-free.

Most previous real-world studies comparing IFX and VDZ used a myriad of clinical and endoscopic endpoints, usually with a follow-up time of up to 1 year, and they have shown conflicting results. Two meta-analyses of several randomized control studies accumulating to over 2700 patients did not find a significant efficacy difference between VDZ and IFX for induction and maintenance of endoscopic remission at 52 weeks (1 year) of follow-up in treatment-naïve patients [10,11]. Conversely, in a large multi-center observational study, patients treated with VDZ were more likely to achieve and maintain steroid-free remission [8] during a similar time frame. In another, smaller, real-world study of 97 patients, the efficacy of VDZ and IFX was comparable in achieving clinical remission at 1 year. Still, VDZ was associated with a higher chance of steroid-free remission [12]. In our cohort, the follow-up period was significantly longer compared to these previously published studies, and we assessed DS as the endpoint.

Studies using DS as an efficacy endpoint are fewer in number. A nationwide Swedish register-based study assessing DS for second-line treatment with IFX and VDZ showed similar DS between these medications [13]. Based on a claims database, a second study from the USA described drug persistence (sustainability) tendencies, with slightly longer drug persistence for IFX, without direct comparison between the drugs and no adjustment to different factors that might affect DS [14]. In a nationwide Korean register-based study, IFX drug persistence was assessed and was longer than other drugs investigated, but the study did not include VDZ [15]. On the other hand, other studies have shown longer DS with VDZ compared to IFX [16,17], similar to our study results.

Several key factors might explain the different results between studies. First, our cohort found that patients treated with IFX and VDZ differed significantly in baseline characteristics, including age and patient-level clinical data indicative of disease severity; this key factor may be unintentionally translated into erroneous results in registry-based and real-world studies lacking sufficient patient-level clinical data. Even propensity score adjustment may be inaccurate without detailed patient-level clinical data. The wide range of clinical data, including key lab data, in our study were adjusted for disease severity, age, and sex, which is a further strength contributing to the confidence of our findings.

In our cohort, in contrast to patients naïve to biologic therapies, there was no difference between the two drugs in biologic-treatment-experienced patients. This was also true for patients treated with VDZ and switched to IFX or vice versa. One possible explanation is that once immunogenicity starts to appear, it targets all biologic drugs alike. Although the antibodies are drug-specific, the final outcome may be similar. This may be supported by studies showing the superiority of combination therapy with an immunomodulator over monotherapy with a biologic [18]. Nevertheless, there are limited data regarding efficacy among biologic-experienced patients, and data, if available, are mainly for anti-TNF-experienced patients, and the results are conflicting. In a multi-center study, VDZ was superior to IFX in achieving steroid-free remission in both treatment-naïve and treatment-experienced patients [8]. In contrast, in the nationwide Swedish register-based study assessing second-line treatment mentioned above, the efficacy of IFX and VDZ at 1 year appeared largely similar [14]. In a recently published post hoc analysis of several UC clinical trials by Narula N et al., IFX had higher rates than VDZ for clinical and endoscopic remission at 1 year [19]. It is difficult comparing previous studies and ours. Very few studies or meta-analyses compare IFX and VDZ in this specific subgroup in terms of sustainability. Previous studies’ follow-up length is mostly limited to 1 year, whereas our study evaluates a much longer follow-up period (up to 300 weeks).

Our study has some limitations. First, our study includes a relatively small number of patients and is a single-center study. Adding to the study’s retrospective nature, this carries a risk of bias. Nevertheless, the large amount of data per patient (including detailed lab parameters) enabled us to overcome these limitations to a certain extent by adjusting according to the different parameters, especially for disease severity. The main limitation, as expected from real-world data, is the lack of a controlled environment. Our endpoint was defined as steroid-free, surgical-free, and hospitalization-free treatment duration without specific documentation of mucosal healing. This necessitates a degree of confidence that the clinical management is following acceptable international guidelines.

## 5. Conclusions

In conclusion, in this population-based study, VDZ showed significantly longer DS in treatment-naïve patients compared to IFX, also following adjustments for disease severity. There was no difference in DS between VDZ and IFX in treatment-experienced patients and patients switching from one drug to the other. This supports prescribing VDZ as a first-line biologic choice in moderate-to-severe UC.

## Figures and Tables

**Table 1:** Basic characteristics of treatment-naïve patients.

Parameter		IFX	VDZ	All	*p*-Value
Male		47 (61%)	60 (55%)	107 (57.5%)	0.454
Disease extent					0.149
Disease extent	Extensive Colitis	28 (36.3%)	31 (28.4%)	59 (31.7%)	0.267
	Left-sided UC	41 (53.2%)	53 (48.6%)	94 (50.4%)	0.555
	Proctitis	8 (10.3%)	24 (22%)	32 (17.2%)	0.048
Combination therapy		11 (14.2%)	3 (2.7%)	14 (7.5%)	0.004
Age at Diagnosis (years median, IQR)		32 (20–48)	32 (21–46)	32 (21–47)	0.869
Age at treatment start (years, median, IQR)		37 (25–55)	42 (28–57)	40.5 (26–56)	0.293
Diagnosis length at treatment start (years, median, IQR)		2 (0–6)	4 (1–12)	2 (1–11)	0.104
Calprotectin (µg/g, median, IQR)		4425 (1350–5870)	529 (161–1339)	890 (196–3230)	0.001
Hemoglobin (g/dL, years, median, IQR)		11 (9.5–12.9)	13.1 (12–14.1)	12.7 (10.8–13.8)	<0.001
Platelets (K/micl, median, IQR)		309 (242–396)	284 (225–366)	298 (231–376)	0.187
Hematocrit (%, median, IQR)		33.8 (30.5–38.9)	40.9 (38–43)	38.8 (33.9–42.5)	<0.001
Hb/Hct ratio (median, IQR)		0.32 (0.31–0.33)	0.32 (0.31–0.33)	0.32 (0.31–0.33)	0.640
Eosinophils (K/micl, median, IQR)		0.1 (0.0–3)	0.2 (0.1–0.8)	0.1 (0.0–3)	0.589
Neutrophils (K/micl, median, IQR)		6 (4.6–9.1)	5.2 (4–7.3)	5.5 (4.1–7.9)	0.203
Lymphocytes (K/micl, median, IQR)		1.4 (1–2)	1.8 (1.3–2.4)	1.6 1.2–2.3)	0.009
Neutrophil-to-lymphocyte ratio (median, IQR)		4.2 (2.7–7)	2.7 (2–4.6)	3.2 (2.2–5.4)	0.000
Leukocytes (K/micl, median, IQR)		9 (6.7–11.3)	7.9 (6.3–10.1)	8.2 (6.3–10.6)	0.497
Albumin (g/dL, median, IQR)		3.4 (2.8–4.1)	4.3 (4.1–4.6)	4.2 (3.5–4.5)	<0.001
Creatinine (mg/dL, median, IQR)		0.6 (0.5–0.9)	0.8 (0.7–0.9)	0.8 (0.6–0.9)	0.002
CRP (mg/dL, median, IQR)		1.3 (0.4–3.5)	0.5 (0.2–0.9)	0.6 (0.2–1.8)	0.002
CPK (U/L, median, IQR)		43 (30–76)	59 (43–115)	53 (31–85)	0.843
Phosphorus (mg/dL, median, IQR)		3.3 (2.9–3.8)	3.4 (2.9–3.8)	3.3 (2.9–3.8)	0.425
Total protein (g/dL, median, IQR)		6.2 (5.4–7)	7.2 (6.9–7.5)	7 (6.3–7.4)	<0.001

UC—Ulcerative colitis, IFX—infliximab, VDZ—vedolizumab, IQR—interquartile range, CRP—C-reactive protein, CPK—creatine phosphokinase.

**Table 2:** Association between the parameters and DS over time.

Parameter		Hazard Ratio (CI 95%)	*p*-Value (Chi-Sq)	Adjusted * HR (CI 95%)	Adjusted * *p*-Value
VDZ (compared with IFX)		0.48 (0.3–0.76)	0.002	0.55 (0.3–0.98)	0.042
Male		0.87 (0.55–1.36)	0.546	1.05 (0.6–1.84)	0.851
Disease extent	Proctitis	0.77 (0.39–1.53)	0.469	—	—
	Left-sided	0.81 (0.5–1.32)	0.412	—	—
	Extensive	1.25 (0.79–1.99)	0.827	—	—
Combination therapy		1.59 (0.09–26.78)	0.352	0.97 (0.44–2.13)	0.939
Age at diagnosis		0.98 (0.97–1.00)	0.119	—	—
Age at treatment start		0.98 (0.97–0.99)	0.032	0.98 (0.97–1)	0.091
Disease length		0.97 (0.95–1)	0.166	—	—
Calprotectin		1 (1–1)	0.070	—	—
Hemoglobin (Hb)		0.89 (0.8–0.99)	0.037	0.73 (0.46–1.16)	0.187
Platelets		1 (1–1)	0.062	—	—
Hematocrit (Hct)		0.96 (0.93–1)	0.051	1.11 (0.94–1.3)	0.195
Hb/Hct Ratio		0 (0–135.09)	0.188	—	—
Eosinophils absolute number		0.38 (0.11–1.31)	0.126	0.39 (0.11–1.36)	0.142
Neutrophils absolute number		1.04 (0.98–1.12)	0.164	—	—
Lymphocytes absolute number		1.29 (0.99–1.68)	0.059	—	—
Neutrophil-to-lymphocyte ratio		0.99 (0.96–1.02)	0.835	0.98 (0.94–1.02)	0.503
White blood count		1.04 (0.98–1.12)	0.164	—	—
Albumin		0.68 (0.52–0.9)	0.007	0.74 (0.44–1.24)	0.603
Creatinine		0.31 (0.11–0.88)	0.028	—	—
CRP		1 (0.94–1.07)	0.821	0.97 (0.9–1.06)	0.603
CPK		0.99 (0.99–1)	0.609	—	—
Phosphorus		1.37 (1.00–1.89)	0.049	—	—
Total Protein		0.77 (0.62–0.97)	0.026	1.07 (0.7–1.64)	0.101

* Adjustment for factors affecting disease severity, including hemoglobin, hematocrit, neutrophil-to-lymphocyte ratio, albumin, CRP, total protein, age and combination therapy with an immunomodulator. HR—hazard ratio, CI—confidence interval, UC—Ulcerative colitis, IQR—interquartile range, CRP—C-reactive protein, CPK—creatine phosphokinase.

## Data Availability

For data requests, please contact Jacob E. Ollech (jacobel@clalit.org.il). Data will be provided only under authorization of Rabin Medical Center according to local patient privacy regulations.

## Supplementary Materials

The following supporting information can be downloaded at: https://www.mdpi.com/article/10.3390/jcm12134488/s1, Table S1: Baseline characteristics of those treated with subsequent second drug; Table S2: Parameters associated with DS of subsequent treatment; Table S3: Baseline differences between patients with long-term follow-up; Table S4: Association between different parameters and long term (>156 weeks) sustainability.
