# Supplementary material for: Vedolizumab Is Associated with Longer Drug Sustainability Compared to Infliximab in Moderate-to-Severe Ulcerative Colitis: Long-Term Real-World Cohort Data
Source: J Clin Med. 2023 Jul 4;12(13):4488. doi: 10.3390/jcm12134488 (PMC10342318; doi:10.3390/jcm12134488)
Supplement: Supplementary file 1 [file jcm-12-04488-s001.zip › jcm-2446575-supplementary.pdf]

*Supplementary file*

**VEDOLIZUMAB IS ASSOCIATED WITH LONGER DRUG SUSTAINABILITY COMPARED TO INFLIXIMAB IN MODERATE-TO-SEVERE ULCERATIVE COLITIS: LONG TERM REAL-WORLD COHORT DATA**

**Table S1.** Baseline characteristics of those treated with subsequent second drug.

| | IFX | VDZ | All | p-value |
|---|---|---|---|---|
| N (%) | 8 (28.5) | 20 (71.4) | 28 (100) | |
| Male (%) | 6 (75) | 10 (50) | 16 (57.1) | 0.401 |
| Extensive Colitis (%) | 4 (50) | 6 (30) | 10 (35.7) | 0.400 |
| Left-sided UC (%) | 2 (25) | 14 (70) | 16 (57.1) | 0.044 |
| Proctitis(%) | 2 (25) | 0 (0) | 2 (7.1) | 0.074 |
| Combination therapy (%) | 2 (25) | 1 (5) | 3 (10.7) | 0.188 |
| Age at diagnosis (years, median, IQR) | 25.5 (18-37) | 39 (21.5-49) | 35.5 (20-46) | 0.101 |
| Age at treatment start (years, median, IQR) | 32.5 (20-46) | 48.5 (30-59.5) | 42 (27.57.5) | 0.108 |
| Disease length (years, median, IQR) | 4 (0.5-8.5) | 5 (1.5-11) | 5 (1-10.5) | 0.794 |
| Calprotectin (µg/g, median, IQR) | 3850 (1100-5570) | 4215 (24-9838) | 4032 (1100-5570) | 1 |
| Hemoglobin (Hb) (g/dL, median, IQR) | 12.8 (10.3-13.8) | 13 (10.8-14) | 12.9 (10.8-14) | 0.710 |
| Platelets (K/micl, median, IQR) | 238.5 (193-409.5) | 273 (234-398) | 271 (217.5-409.5) | 0.795 |
| Hematocrit (Hct) (%, median, IQR) | 40.5 (34-43.7) | 39.7 (34.7-40.8) | 39.8 (34.6-41.3) | 0.645 |
| Hb/Hct ratio (median, IQR) | 0.3 (0.3-0.3) | 0.3 (0.3-0.3) | 0.3 (0.3-0.3) | 0.014 |
| Eosinophils (K/micl, median, IQR) | 0.2 (0.1-0.5) | 0.2 (0.1-.3) | 0.2 (0.1-0.3) | 0.353 |
| Neutrophils (K/micl, median, IQR) | 6.15 (5-6.9) | 5.5 (3.9-7.7) | 5.7 (4-7.3) | 0.917 |

| | | | | |
|---|---|---|---|---|
| Lymphocytes (K/micl, median, IQR) | 2.5 (2.3-4.1) | 2 (1.6-2.4) | 2.3 (1.6-2.5) | 0.037 |
| Neutrophil-to-lymphocyte ratio (median, IQR) | 2.2 ((1.5-2.5) | 2.8 (2.1-4.3) | 2.4 (1.8-3.5) | 0.088 |
| Leukocytes (K/micl, median, IQR) | 10 (8.1-12.6) | 8.3 (7.1-10.2) | 9 (7.2-11.1) | 0.281 |
| Albumin (g/dL, median, IQR) | 4.1 (3.6-4.5) | 4.1 (4-4.4) | 4.1 (3.9-4.4) | 0.912 |
| Creatinine (mg/dL, median, IQR) | 0.9 (0.8-1) | 0.7 (0.6-0.8) | 0.7 (0.6-0.9) | 0.265 |
| CRP (mg/dL, median, IQR) | 0.3 (0.1-1.1) | 0.5 (0.2-1.9) | 0.5 (0.2-1.8) | 0.263 |
| CPK (U/L, median, IQR) | 95 (75-115) | 53 (41-69) | 55 (42-75) | 0.527 |
| Phosphorus (mg/dL, median, IQR) | 3.6 (3.1-4.1) | 3.7 (3.3-3.9) | 3.7 (3.3-3.9) | 0.601 |
| Total protein (g/dL, median, IQR) | 7.3 (6.4-7.8) | 7.3 (6.7-7.8) | 7.3 (6.7-7.8) | 0.502 |

UC – Ulcerative colitis, IQR – interquartile range, CRP – C-reactive protein, CPK – creatine phosphokinase.

**Table S2.** Parameters associated with DS of subsequent treatment.

| Parameter | | HR (95% CI) | p-value | Adjusted* HR (95% CI) | Adjusted* p-value |
|---|---|---|---|---|---|
| Drug - VDZ | | 0.3 (0.1-1.3) | 0.145 | 0.0 (0.0-14.7) | 0.316 |
| Male | | 1.6 (0.4-5.4) | 0.410 | 446.4 (5.3-36943) | 0.006 |
| Disease extent | Extensive colitis | 1.4 (0.4-4.5) | 0.533 | -- | -- |
| | Left sided colitis | 0.69 (0.2-2.1) | 0.527 | -- | -- |
| | Proctitis | 1.6 (0.2-10.2) | 0.579 | -- | -- |
| Combination therapy | | 0.8 (0.1-5.1) | 0.865 | 0.0 (0.0-0.0) | 0.0053 |
| Age at diagnosis | | 0.9 (0.9-0.9 | 0.032 | -- | -- |
| Age at treatment start | | 0.9 (0.9-1) | 0.064 | 0.8 (0.7-0.9) | 0.026 |
| Length of diagnosis | | 1 (0.9-1.1) | 0.750 | -- | -- |
| Calprotectin | | 1 (0.0-2.3E) | 0.999 | -- | -- |
| Hemoglobin(Hb) | | 0.7 (0.5-1) | 0.072 | 0.26 (0.0-9.6) | 0.465 |
| Hematocrit (Hct) | | 0.9 (0.8-1) | 0.261 | 0.9 (0.2-4) | 0.944 |
| Hb/Hct ratio | | 0.0 (0.0-0.9) | 0.000 | -- | -- |
| Platelets | | 1 (1-1) | 0.059 | -- | -- |
| Leukocytes | | 1.1 (1-1.3) | 0.035 | -- | -- |
| Eosinophils | | 1.4 (0.0-30) | 0.792 | 0.0 (0.0-0.0) | 0.001 |
| Neutrophils | | 1.1 (0.9-1.3) | 0.087 | -- | -- |
| Lymphocytes | | 1.9 (0.9-3.7) | 0.0502 | -- | -- |
| Neutrophil-to-lymphocyte ratio | | 1.1 (0.7-1.6) | 0.549 | 3.5 (0.6-19.8) | 0.148 |
| Albumin | | 0.4 (0.1-1.8) | 0.265 | 0.0 (0.0-0.3) | 0.025 |
| Creatinine | | 0.6 (0.0-9.3) | 0.778 | -- | -- |
| CRP | | 0.9 (0.6-1.2) | 0.618 | 0.1 (0.0-1.7) | 0.138 |
| CPK | | 1 90.9-1) | 0.303 | -- | -- |
| Phosphorus | | 4.5 (1.4-14.5) | 0.011 | -- | -- |
| Total protein | | 1.7 (0.5-5.5) | 0.328 | 1207.9 (6.4-22E) | 0.007 |

*Adjustment for factors affecting disease severity, including hemoglobin, hematocrit, neutrophil-to-lymphocyte ratio, albumin, CRP, total protein, age and combination therapy with an immunomodulator. UC – Ulcerative colitis, IQR – interquartile range, CRP – C-reactive protein, CPK – creatine phosphokinase.

**Table S3.** Baseline differences between patients with long-term follow-up.

| Parameter | | Infliximab | Vedolizumab | ALL | p-value |
|---|---|---|---|---|---|
| N (%) | | 49 (100) | 33 (100) | 82 (100) | |
| Male (%) | | 31 (63.2) | 17 (51.5) | 48 (58.5) | 0.362 |
| Combination therapy (%) | | 6 (12.2) | 1 (3) | 7 (8.5) | 0.233 |
| Disease extent | | | | | 0.208 |
| | Extensive colitis (%) | 14 (28.5) | 9 (27.2) | 23 (28) | 1 |
| | Left-sided UC (%) | 29 (59.1) | 15 (45.4) | 44 (53.6) | 0.263 |
| | Proctitis (%) | 6 (12.2) | 9 (27.2) | 15 (18.2) | 0.143 |
| Age at diagnosis (years, median, IQR) | | 36 (22-49) | 31 (20-54) | 35.5 (21-51) | 0.990 |
| Age at treatment start (years, median, IQR) | | 43 (26-56) | 45 (28-59) | 44 (26-57) | 0.543 |
| Length of diagnosis at treatment start (years, median, IQR) | | 2 (1-5) | 4 (1-11) | 2 (1-10) | 0.212 |
| Calprotectin (median, IQR) | | 5714 (1114-7011) | 575 (255-1197) | 1114 (378-5714) | 0.111 |
| Hemoglobin (median, IQR) | | 11.3 (10.4-13.2) | 12.9 (11.6-13.9) | 12.4 (10.8-13.5) | 0.001 |
| Platelets (median, IQR) | | 309 (260-393) | 269 (208-339) | 301 (231-376) | 0.05 |
| Hematocrit (median, IQR) | | 35 (32-39.9) | 39 (36.4-42.7) | 37.8 (33.5-42.3) | <.001 |
| Hb/Hct ratio (median, IQR) | | 0.3 (0.3-0.3) | 0.3 (0.3-0.3) | 0.3 (0.3-0.3) | 0.701 |
| Eosinophils (median, IQR) | | 0.1 (0.0-0.2) | 0.3 (0.1-0.4) | 0.2 (0.1-0.3) | 0.001 |
| Neutrophils (median) | | 6 (4.5-8.4) | 4.8 (4-6.2) | 5.6 (4.2-7.8) | 0.434 |
| Lymphocytes (median, IQR) | | 1.3 (1-2) | 2 (1.3-2.4) | 1.6 (1.1-4) | 0.069 |
| Neutrophil-to-lymphocyte ratio (median, IQR) | | 4.3 (2.7-5.7) | 2.4 (2-3.1) | 3.3 (2.2-5.5) | 0.233 |
| Leukocytes (median, IQR) | | 8.9 (7.1-10.8 | 7.9 (6.5-9.7) | 8.4 (6.5-10.3) | 0.629 |
| Albumin (median, IQR) | | 4 (3.1-4.2) | 4.4 (4-4.5) | 4.1 (3.4-4.5) | <.001 |
| Creatinine (median, IQR) | | 0.7 (0.6-0.9) | 0.8 (0.6-0.9) | 0.7 (0.6-0.9) | 0.127 |
| CRP (median, IQR) | | 1.6 (0.4-3.7) | 0.4 (0.2-0.9) | 0.7 (0.2-2.2) | 0.013 |
| CPK (median, IQR) | | 48 (30-94) | 54 (38-69) | 51 (34-85) | 0.965 |
| Phosphorus (median, IQR) | | 3.4 (3-3.8) | 3.4 (2.9-3.8) | 3.4 (3-3.8) | 0.717 |
| Total protein (median, IQR) | | 6.6 (5.6-8.1) | 7.2 (6.9-7.7) | 7 (6.4-7.5) | 0.000 |

**Table S4.** Association between different parameters and long term (>156 weeks) sustainability.

| parameter | Odds Ratio (CI 95%) | p-value (Chi-Sq) | Adjusted odds ratio (CI 95%) | Adjusted p-value |
|---|---|---|---|---|
| Vedolizumab vs Infliximab | 18.1 (4.3-75.4) | <.000 | 67 (6-741.9) | 0.000 |
| Gender (male) | 0.84 (0.3-2.0) | 0.708 | 1.1 (0.2-5.4) | 0.862 |
| Left sided UC vs. Extensive colitis | 1.2 (0.4-3.3) | 0.700 | -- | -- |
| Proctitis vs. Extensive colitis | 1.9 (0.4-8.0) | 0.334 | -- | -- |
| Combination therapy | 0.4 (0.0-2.1) | 0.305 | 0.6 (0.0-4.7) | 0.661 |
| Age at diagnosis | 0.9 (0.9-1) | 0.785 | -- | -- |
| Age at treatment start | 0.9 (0.9-1) | 0.916 | 0.9 (0.9-1) | 0.627 |
| Length of disease (years) | 1 (0.9-1) | 0.845 | -- | -- |
| Calprotectin | 1 (0.9-1) | 1 | -- | -- |
| Hemoglobin | 1.1 (0.93-1.4) | 0.167 | 1.5 (0.3-6.3) | 0.568 |
| Platelets | 0.9 (0.9-1) | 0.098 | -- | -- |
| Hematocrit | 1 (0.9-1.1) | 0.168 | 0.7 (0.4-1.3) | 0.367 |
| Hb/Hc ratio | 999.9 (0.0-999.9) | 0.612 | -- | -- |
| Eosinophils absolute number | 6 (0.4-88.6) | 0.191 | 0.3(0.0-43.7) | 0.669 |
| Neutrophils absolute number | 0.9 (0.7-1) | 0.211 | -- | -- |
| Lymphocytes absolute number | 0.9 (0.5-1.6) | 0.935 | -- | -- |
| Neutrophil-to-lymphocyte ratio | 1 (0.9-1) | 0.999 | 1 (0.9-1.2) | 0.338 |
| White blood count | 0.9 (0.7-1) | 0.190 | -- | -- |
| Albumin | 2.1 (1-4.2) | 0.030 | 6.9 (0.4-116.9) | 0.180 |
| Creatinine | 1.4 (0.3-5.3) | 0.576 | -- | -- |
| CRP | 0.9 (0.8-1) | 0.492 | 1 (0.8-1.2) | 0.891 |
| CPK | 0.9 (0.9-1) | 0.305 | -- | -- |
| Phosphorus | 0.4 (0.2-1) | 0.055 | -- | -- |
| Total protein | 1.5 (0.9-2.4) | 0.088 | 0.4 (0.0-1.9) | 0.267 |
